# Electrical and Electromagnetic Geophysical Prospecting for the Monitoring of Rock Glaciers in the Dolomites, Northeast Italy

**DOI:** 10.3390/s21041294

**Published:** 2021-02-11

**Authors:** Mirko Pavoni, Fabio Sirch, Jacopo Boaga

**Affiliations:** Department of Geosciences, University of Padova, via Gradenigo 6, 35131 Padova, Italy; mirko.pavoni@phd.unipd.it (M.P.); fabio.sirch@studenti.unipd.it (F.S.)

**Keywords:** ERT, FDEM, EMI, frequency domain electro-magnetometry, mountain permafrost, rock glacier, Dolomites

## Abstract

The monitoring of rock glaciers plays a relevant role in relation to natural hazards in high mountain environments. Due to the climate warming, mountain permafrost is thawing, and its degradation is influencing the triggering and the evolvement of processes such as rockfalls, landslides, debris flows and floods. Therefore, the study and monitoring of these periglacial forms have both a scientific and economic importance. We tested electrical and electromagnetic measurements along the same investigation lines, in two different sites of the Dolomites area (Northeast Italy). Electrical prospecting exploits the high resistivity contrast between frozen and non-frozen debris. However, these measurements have high logistic demands, considering the complex rock glaciers surface and the need of ground galvanic contact. For this reason, we tried to compare electrical measurements with electromagnetic contactless ones, that theoretically can be used to define the distribution of electrical resistivity in the first subsoil in a quicker and easier way. The obtained results show that the joint use of the two methods allows us to characterize a rock glacier subsoil with good confidence. Finally, the advantages and disadvantages of both the techniques are discussed.

## 1. Introduction

Permanent frozen soils and rock glaciers are important components of the high mountain eco-system, occurring at a variety of locations worldwide [1]. In general terms, permafrost is the part of soil that remains continuously below 0 °C for two or more years [2]. Among permafrost morphologies, the rock glaciers consist of coarse surface debris that insulates an ice core or an ice-debris mixture. These geomorphological landforms have creeps along the slopes and can present various rates of movement, from 0 to a few meters per year, and are characteristically serving as a visible indicator of mountain permafrost [3,4,5,6]. However, in contrast to lowland permafrost, the distribution and thawing of mountain permafrost is spatially more heterogeneous and complex, according to the strong variability of topography, geomorphology, and climate conditions in the Alps [7]. Moreover, alpine permafrost is often not directly visible in the landscape. Therefore, the analysis of permafrost distribution and degradation, which is a relevant proxy of the climate change and global warming, are subject to greater uncertainties in comparison to the observable glaciers [8].

The internal structure and temperature of rock glaciers and frozen soils can be studied using direct borehole logging [4,9,10,11]. Borehole measurements provide valuable and reliable information on ground temperature evolution and changes at various depths [12]. However, drilling in high mountain ice-rich ground is challenging and expensive, and the boreholes provide only punctual temperature information [13]. To obtain more extensive data, beyond the single measured point, geophysical surveys have been frequently applied [14,15,16,17,18]. Since soil physical properties, such as electrical resistivities (or conductivities), dielectric properties and seismic wave velocities, vary greatly in the case of phase change between water and ice, geophysical techniques, such as electrical resistivity tomography (ERT), ground-penetrating radar (GPR) and refraction seismic tomography (RST), have been intensively used in these environments [19,20,21,22]. Geophysical prospecting that is sensible to ice presence has been used to investigate the subsurface properties of rock glaciers in several study cases, from the characterization of the mountain permafrost to the monitoring of the frozen soil evolution over time [23,24,25,26,27,28,29,30]. In particular, the electrical resistivity tomography (ERT) was applied to provide indirect information about the changes in the ice/water ratio over time and to estimate the active layer thickness (ALT) [31].

The active layer is the superficial layer of permafrost that thaws periodically during the hot season. The thickening of the active layer means the loss of the internal ice, which acts as a stabilizing binding element for the debris. This implies a reduced mechanical stability of the rock glacier, increasing the availability of erodible material [32]. Degrading permafrost in alpine environments can lead to a variety of hazards such as debris flows, rock falls or outburst floods from breeching of proglacial lakes [33]. Rising temperatures and ice volumes changes in the ground during the past two decades have likely caused a strong acceleration of rock glacier movement and degradation [34,35,36,37,38,39]. The potential accelerations induced by intense climate warming could even make this process worse [12] and, in some cases, a very strong and non-reversible acceleration, up to decameters per year, was observed [8]. Moreover, the thawing process can generate rockfalls and debris flow activity at the rock glaciers front [40,41,42,43], or even rupture and collapse of the entire front [44]. Thermokarst lakes, which can develop on the surface of the rock glacier during the melt season, are a further source of danger. Outbursts of a thermokarst lake occurred at Gruben rock glacier (Switzerland) in 1968 and 1970, causing flooding and debris flows [45,46]. Furthermore, the decrease in ice in the ground, linked with an increase in meltwater, can strongly influence the stability and bearing capacity of the infrastructure foundations [47,48]. This is particularly challenging in the Dolomites area, Northeastern Alps of Italy, where the high mountain environment is densely populated. Dolomites are in fact a UNESCO world heritage protected environment, visited by millions of tourists every year (www.dolomitiunesco.info (accessed on 11 February 2021)). The Dolomites mountains host many touristic activities such as hiking, alpinism, and skiing, and many infrastructures such as hotels, ski resorts, cable cars, traffic roads, and mountain huts. For all these reasons, the dynamics of the rock glaciers have a relevant socio-economic impact, and mountain permafrost monitoring is one of the key aspects for the management of natural hazards in the Dolomites area. Therefore, reliable geophysical methods to characterize alpine rock glaciers have both an economic and scientific interest.

In this paper, we present the use of electrical (ERT) and frequency-domain electromagnetic (FDEM) methods to characterize two rock glaciers of the Sella Group mountains in the Dolomites. As discussed, the ERT technique has historically been used to study mountain permafrost and rock glaciers, with excellent results in several monitoring programs in the Alps [12,49]. However, ERT technique needs heavy equipment and requires the use of multiple steel electrodes with good galvanic contact with the substrate. Problems of contact resistance between the electrodes and rocky ground surface are common, and in some cases solved by adding salt-water between electrodes and boulders [50]. As a matter of fact, ERT surveys, despite the great potential, are not easy and fast to be executed in this kind of environment. Thus, usually we are not able to perform many investigation lines and, as the results are 2D resistivity sections, it is difficult to obtain enough information to completely characterize a heterogeneous environment such as a rock glacier. On the other hand, FDEM is a promising geophysical technique [51] that is very fast to perform since no galvanic contact with the ground is needed to assess the electrical properties of the subsoil. The FDEM technique has lower resolution and depth penetration if compared to ERT but has the potential for a quick characterization of larger areas even if, due to the intrinsic limited resolution, it should be considered as an integration rather than an alternative to the ERT method for rock glacier studies. The present work confirmed the ability of the FDEM technique to define the presence of ground ice and estimate the ALT, as recently tested on the Schaftberg rock glacier in the Swiss Alps [13]. We performed ERT and FDEM surveys in two different rock glaciers of the Sella Group mountains, comparing the results of the two techniques, performed on the same investigation line. We firstly introduce the study site, ERT and FDEM techniques, and finally we discuss the obtained results, highlighting limits and advantages of both the geophysical surveys.

## 2. Site Description

**Figure 1:**
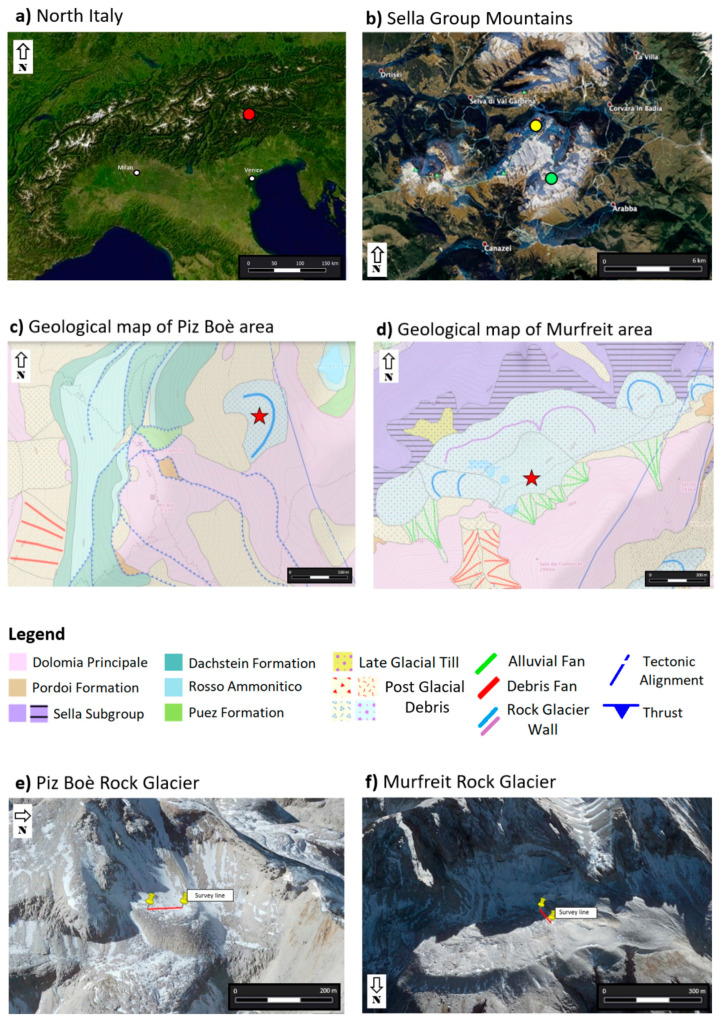
(**a**) The position of Sella Group mountains in Northeastern Italy (red dot); (**b**) the location of the Murfreit (yellow dot) and Piz Boè (green dot) rock glaciers in the Sella Group mountains; (**c**) geological map of the Piz Boè area (red star is the survey position); (**d**) geological map of the Murfreit area (red star is survey position); (**e**) the survey line performed on the Piz Boè rock glacier (red line); (**f**) the survey line executed on the Murfreit rock glacier (red line).

Our surveys were performed in the summer of 2020, on the Murfreit and Piz Boè active rock glaciers in the Sella Group mountains, which is part of the Dolomites chain in Northeastern Italy (Figure 1a). Murfreit develops on the northside of Sella Group mountains (Figure 1b), it is an ice-cored lobate rock glacier of 420 m length, 1100 m width and it covers an area of 0.34 km^2^. The front, composed of steep slopes, terminates at an elevation of 2590 m and the rooting zone is at 2770 m. The rock glacier is bordered by a steep wall on the southside, which reaches an elevation of about 3000 m and it is composed by the “Dolomia Principale” formation, strongly jointed and fractured by several steep faults (see Figure 1d). Consequently, the rock glacier is entirely made up of dolomite debris of a grain size varying between 1 cm and 100 cm, even if at the surface layer the sizes are mainly of 1–10 and 11–20 cm [42]. The investigation line has been realized longitudinally to the development of the rock glacier, for a total length of 70.5 m (see Figure 1f).

On the contrary, the investigation line performed on the rock glacier of Piz Boè was defined orthogonally to its development (Figure 1e). Piz Boè is located on the Southeast side of Sella Group mountains (see Figure 1b) and the rock glacier, or rather the debris-covered glacier [52], has a much lower extension than Murfreit. It has also a lobate shape, but with a width of 180 m and a length of 220 m, for a total extension of 0.014 km^2^. This rock glacier develops in the area facing the Piz Boè peak (3152 m), at an average altitude of about 2900 m, and its bordered by a step wall on the southwest side. The wall is mainly composed by the “Dolomia Principale” formation and by limestones of the “Dachstein’’ formation, cut by a system of low angle thrust (see Figure 1c). Therefore, the debris of this rock glacier has a calcareous and dolomite lithology, and it is mainly composed of decimetric up to metric size angular blocks.

## 3. Methods

### 3.1. Electrical Resistivity Tomography (ERT)

Electrical methods are probably the most widely used near-surface geophysical techniques for environmental investigations [53]. Based on the number of scientific publications in the past decades and the large variety of applications, ERT is maybe the most universally applicable geophysical method for research in mountain permafrost environments [50]. The investigations are realized with an array of dozens of electrodes, by injecting the current *I* in two electrodes (called A–B) and by recording the potential difference ΔV that arise at the other pairs of the electrodes (called M–N). The penetration depth of the current depends mainly on the electrical resistivity of the subsurface, the spacing and the configuration of the electrodes [54]. Using a multi-electrodes system, the measurement can be performed along the entire array and this allows us to retrieve the apparent resistivities *ρ_a_* (Ωm) of the subsoil in a 2D image, called a pseudo-section. The resolution of the survey depends mainly on the distance between electrodes: lower is the spacing and higher is the resolution. The maximum investigation depth, obtained at the center of the line, is linked to the electrode array length and site characteristics [53]. Since the number of electrodes is limited, the length of the electrode line must be determined according to the number of electrodes available and the required resolution. There are different kinds of configurations that can be used to create the quadrupole of measurement, with current electrodes A and B outside the potential electrodes M and N (Wenner-Sclumberger), or the current electrode dipole A–B adjacent to the potential dipole M–N (Dipole–Dipole). The various configurations have advantages and disadvantages, and the choice should be based on the intended application and expected signal strength [53].

**Figure 2:**
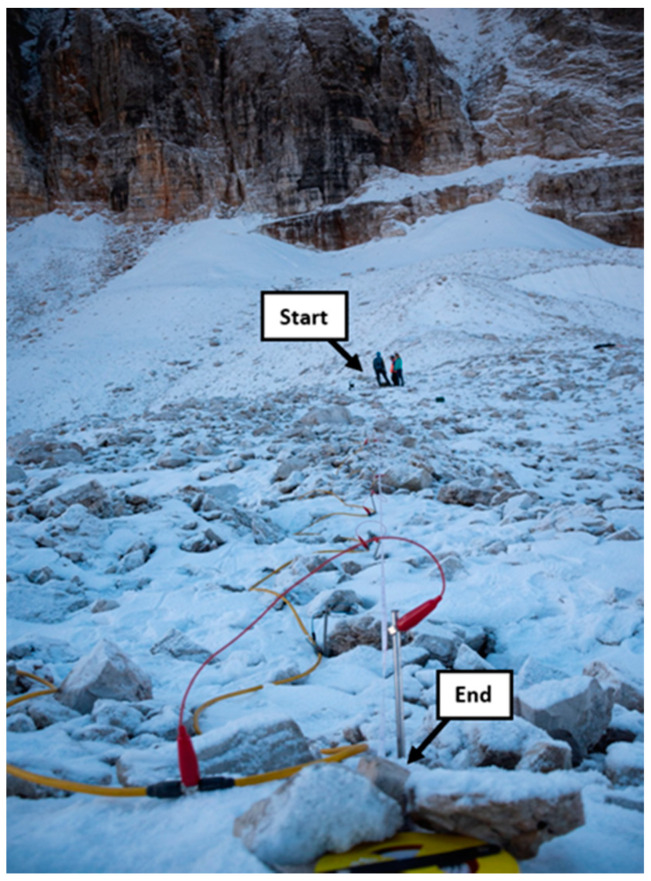
Picture taken during the electrical resistivity tomography (ERT) measurements performed on the Murfreit rock glacier in July 2020. “Start” is the position of the first electrode and “end” is the position of the last electrodes. The line of 70.5 m is composed of 48 electrodes spaced 1.5 m apart.

For the measurements performed on the Murfreit rock glacier in July 2020 (Figure 2/Table 1 for all the acquisition parameters), we used an MAE geophysics digital georesistivimeter (www.mae-srl.it (accessed on 11 February 2021)) and an array of 48 electrodes, spaced 1.5 m apart. Electrodes were hammered into the rocky ground surface, and the galvanic contact has been improved with the addition of salt-water, about 0.3 l for each electrode. In the dry condition, the measured contact resistances had values higher than several hundred of kΩ but the wet condition guaranteed values lower than 50 kΩ, allowing a greater signal-to-noise ratio. We used the Dipole–Dipole skip 0 configuration, acquiring both reciprocal and direct measurements [55], i.e., exchanging current and potentiometric electrodes for each quadrupole of measurement. This way, we are able to find an estimation of the experimental error for the collected dataset. The survey was also performed by applying the stacking procedure, i.e., collecting each quadrupole several times and averaging the results. This also allow us to improve the signal-to-noise ratio because random noise is averaged out. 

Since measured resistivities are influenced by the contribution of different materials that are present in the ground—for this reason they are defined as apparent—the pseudo-section is then inverted adopting codes that, starting from a discretization grid, search the ground model iteratively, which minimizes the misfit between the measured dataset and the synthetic calculated data [54]. In our study cases, firstly we deleted all the measurements that had a potential difference ΔV lower than 1 mV (instrument resolution limit), afterwards we checked the dataset in terms of direct and reciprocal deviation, saving only the quadrupoles with less than 10% of discrepancy, and we performed the inversion process adopting the same error threshold. We used the code R2 based on Occam’s inversion method [53], obtaining an inverted resistivity section with final RMS (Root Mean Square, a mean to evaluate the misfit between measured and calculated datasets) lower than 1 in 3 iterations.

In the study case of Piz Boè (September 2020), the ERT survey was performed with the same routine as in the Murfreit rock glacier (see Table 1), but the acquired dataset was noisier (<50% of quadrupoles saved after the reciprocity check applying a threshold of 20%). For this reason, we preferred to perform the inversion process with the ResIPy code [56], a Python GUI for R2, using all the direct and reciprocal measurements to calculate a power law error model [57,58]. Once again, we removed the measurements with potential difference ΔV lower than 1 mV and, after the data processing, we obtained again an inverted resistivity section with an RMS misfit lower than 1 in 2 iterations.

### 3.2. Frequency Domain Electro-Magnetometry (FDEM)

The electro-magnetometry technique in the frequency domain (hereinafter FDEM) has been widely applied in the environmental application [59] to rapidly map the apparent conductivity of subsoils. Recently, several inversion codes have allowed us to assess a real conductivity model from the raw data [60,61] and, although the FDEM inversion process never gives so detailed and accurate results as ERT, it is useful for many cases of investigation, e.g., salinity [62], water content [63], soil texture [64,65], soil organic matter [66,67], and it has been also widely used in studies of lowland arctic permafrost [68,69,70]. In the mountain permafrost, the resistivity range is very high (10^3^ Ωm–10^6^ Ωm), making the response close to the resolution limit of FDEM instruments (~0.1 mS/m). This is mainly the reason why the technique is not particularly popular in the mountain terrain compared to the arctic environments, where the subsoils are usually more conductive thanks to the large fraction of organic material, the high unfrozen water content during the summer season and the saline conditions in coastal regions [50].

**Figure 3:**
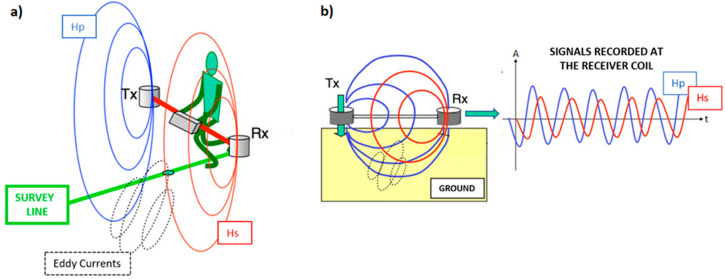
(**a**) Schematic representation of an frequency-domain electromagnetic (FDEM) device. A transmitter coil (Tx) emits a transient primary magnetic field (Hp—blue line) that induces eddy currents (dotted lines) in the ground. These eddy currents generate a secondary electromagnetic field (Hs—red line). (**b**) Hp and Hs are recorded by the receiver coil (Rx). Note that Hp and Hs have amplitude and phase lag [59].

The FDEM method applies Maxwell’s equations to obtain information about the electrical conductivity distribution in the ground [60]. As schematically shown in Figure 3, the device has a transmitter coil (Tx) where an alternating current of frequency *f* (in the order of kHz) flows, generating a primary electromagnetic field (Hp). The latter induces eddy currents in the subsoil which, in turn, generate a secondary electromagnetic field (Hs). Hp and Hs are both measured by the receiver coil (Rx). The ratio between Hs and Hp is a complex number composed by an in-phase component (P) and an out-of-phase, or quadrature, component (*Q*). The measured ratio Hs/Hp is in relationship with the operating frequency, coil separation and orientation, but also on the ground properties: magnetic, conductive and dielectric. Usually, at the frequencies of kHz, dielectric properties can be ignored and, considering that most of the subsoils are practically non-magnetic, the magnetic permeability *μ* of the ground is assumed to be equal to that of free space (*μ*_0_ = 1.257 × 152 10−8 H/m) [60]. Hence, from the quadrature component of the ratio Hs/Hp, the electrical conductivity of the soil can be assessed. This is true only in the so-called “Low Induction Number” (LIN) conditions [59], i.e., when the induction number *β* is <<1:

(1) $$\beta =s\sqrt{\frac{2}{\omega \mu_{0}\sigma \;}}\;$$

where *σ* is the conductivity of the soil, *ω* is the angular frequency (*ω* = 2*πf*) of the signal and *s* is the separation of the two coils. If this condition is verified, we can calculate the apparent conductivity *σ*_a_ of the ground:

(2) $$\sigma_{a}=\;\frac{4}{\omega \mu_{0}s^{2}}Q\;$$

Thanks to the very low electrical conductivities of the high mountain permafrost environment, the LIN condition is practically always satisfied. On the other hand, in this kind of environment, the magnetic field decays rapidly, restricting the penetration depth [50]. To go deeper, without breaking the LIN condition, a lower frequency signal can be used or the distance between the coils can be increased [13].

**Figure 4:**
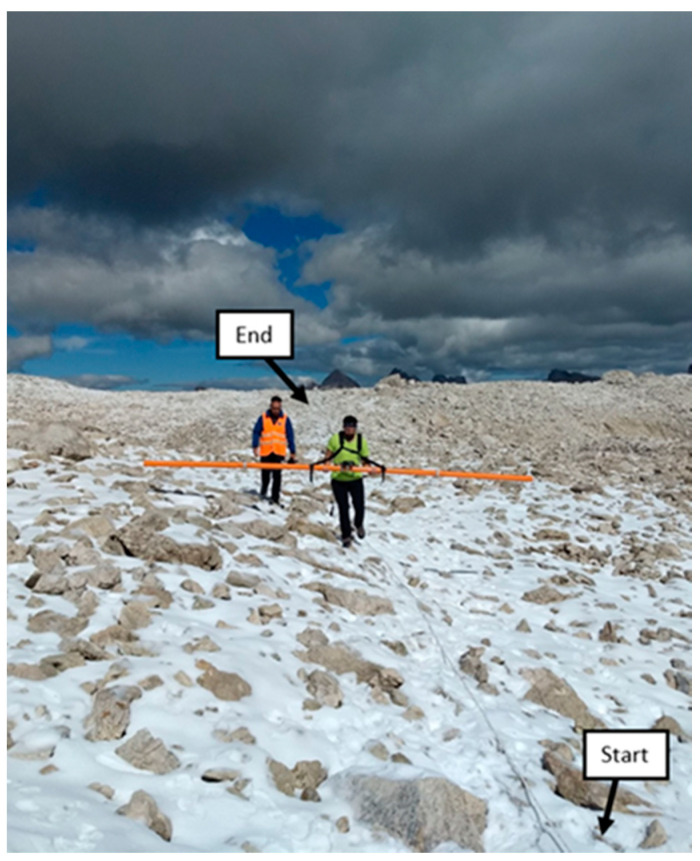
Picture taken during the FDEM measurements performed on the Murfreit rock glacier. “Start” was the position of the first electrode and “end” the position of the last electrode. Note that the measured FDEM transect is the same where the ERT survey was previously executed.

In our study cases, we carried out the FDEM measurements at the rock glaciers of Sella Group mountains on the same investigated lines of the ERT surveys, using a CMD-Explorer probe by Gf-instruments (www.gfinstruments.cz (accessed on 11 February 2021)). The device has an accuracy of ~0.1 mS/m, three different pairs of coils separated by 1.48 m, 2.82 m and 4.49 m, respectively, and a fixed frequency of 10 kHz. In each investigation line, we collected two datasets, keeping an average height of the device as constant as possible to 1 m (Figure 4), one using a horizontal orientation of the coils (*HCP*) and the other with a vertical orientation (*VCP*). We acquired several hundreds of measurement points for each transect, using a sampling step of 0.5 s, collecting the survey a few times to avoid the possible air temperature drift [71,72]. Scattered values, local anomalies or negative values of conductivity may be recorded due to FDEM electronic instrumental drift. However, in our study cases, these potential drift problems only slightly affected the shortest probe (1.48 m) and consequently only the uppermost part of the ground. On the other hand, the longer probes (2.82 m and 4.49 m), allowing deeper investigation depths (see Table 2), are practically unaffected by the scattering [13].

The combination of 3 pairs of coils and horizontal/vertical co-planar modes allows us to have six penetration depths for each point of measurement, and therefore 6 different apparent conductivities. As for the apparent resistivities of ERT, the measured conductivities of FDEM surveys are apparent since they are influenced by the contribution of the different materials that are present in the subsoil. McNeill [59] proposed cumulative sensitivity (CS) functions to describe the relative contribution of materials, below a specific depth, to the measured apparent conductivity. The normalized sensitivities (*R)* for the vertical coils’ position (*VCP*) and horizontal coils’ position (*HCP*) are:

(3) $$R_{VCP}\left(z\right)=\;\sqrt{\left(4z^{2}+1\right)}\;-\;2z$$

(4) $$R_{HCP}\left(z\right)=\;\frac{1}{\sqrt{4z^{2}+1}}\;$$

where *z* is the depth normalized by the coil separation. Equations (3) and (4) say that measurements acquired with coils in the *VCP* mode are more sensitive to the shallow subsurface while measurements acquired in *HCP* mode are more sensitive to deeper depths (see also Table 2). In our study cases, as we acquired the measurements by holding the probe at an average height of 1 m (see Figure 4), the sensitivity patterns may be a little shifted [73]. Since the ALT in the Sella Group mountain sites was expected to be several meters, we focused on the *HCP* mode collected data. The datasets were inverted with the code EMagPy [60], using the CS forward model solution and the L-BFGS-B (Broyden–Fletcher–Goldfarb–Shanno) optimization method [74] to minimize the total misfit between observed values and predicted values from the forward model solution. In order to favor a smoother inversion process, we removed the few anomalous negative values and data outliers (values >2 standard deviations) from the entire dataset. We also applied a smoothing window filter, replacing each data point with the average of the neighboring data points [75]. EMagPy has capabilities to perform quasi-2D inversions, generating inverted FDEM profiles in each point of measurement, which in our cases were very close to each other (on average, 1 measurement every 0.3 m along the transects) given the small sampling step. Before the inversion process, we defined profiles of 20 layers with thickness of 0.4 m. Afterwards, the code calculated a value of conductivity in each layer and profile of the transect, holding a final RMS misfit lower than 1. Finally, we interpolated the individual inverted profiles with the kriging method [76], obtaining pseudo 2D conductivity sections comparable with the corresponding ERT inverted resistivity sections.

## 4. Results

**Figure 5:**
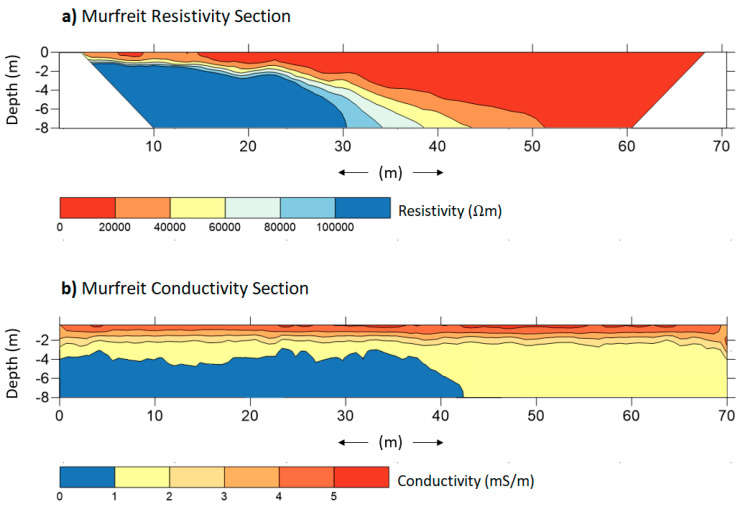
Inverted (**a**) resistivity and (**b**) conductivity sections obtained with ERT and FDEM surveys performed with an investigation line longitudinal to the development of the Murfreit rock glacier.

**Figure 6:**
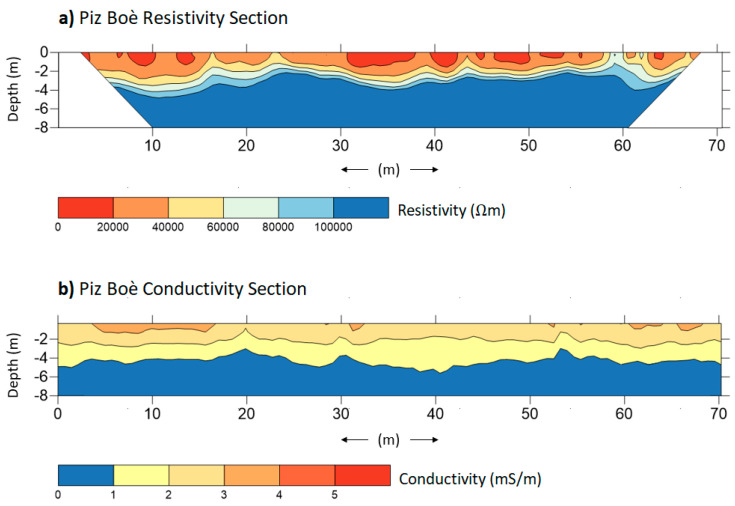
Inverted (**a**) resistivity and (**b**) conductivity sections obtained with ERT and FDEM surveys performed with an investigation line orthogonal to the development of the Piz Boè rock glacier.

Figure 5 and Figure 6 show the results of the investigations performed, respectively, on the Murfreit and Piz Boè rock glaciers. The figures present the inverted (a) resistivity (b) and conductivity sections obtained from the datasets collected, respectively, with ERT and FDEM surveys.

**Figure 7:**
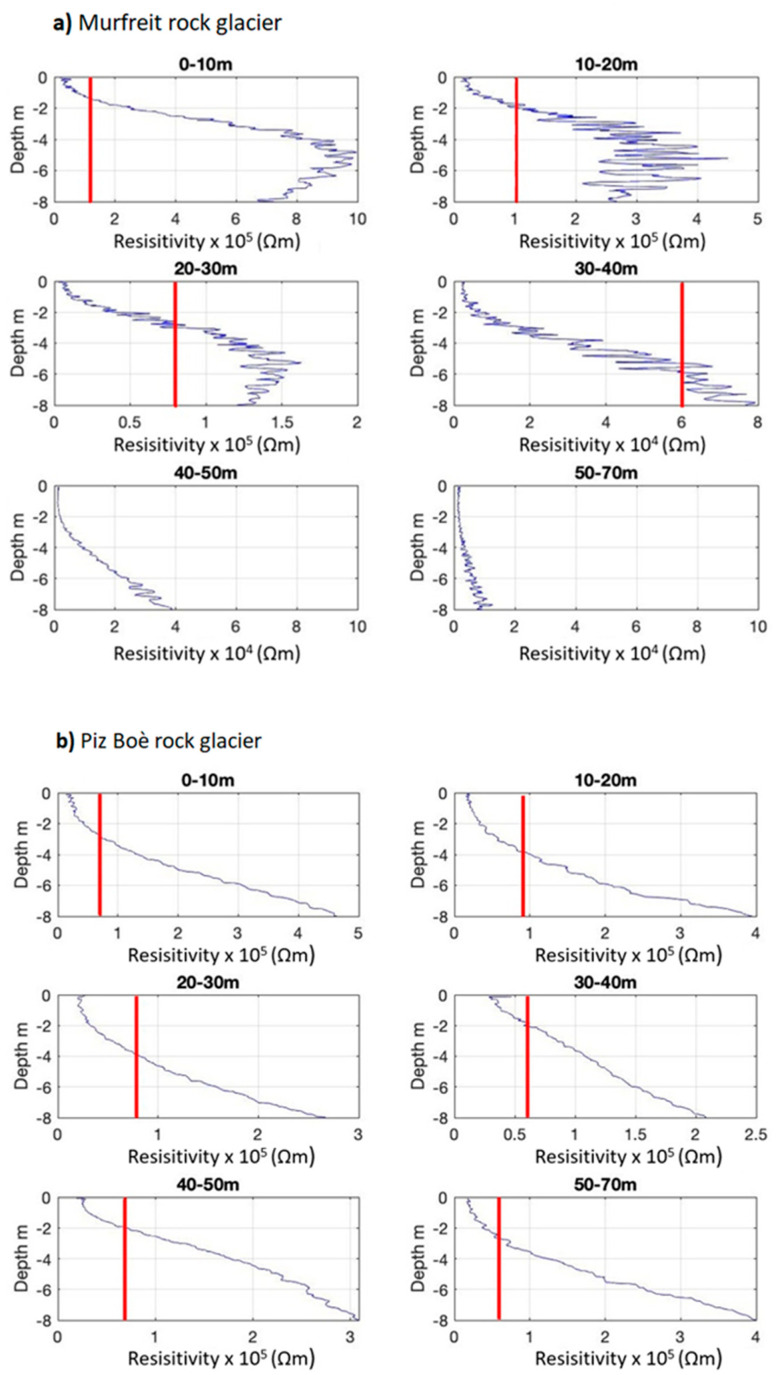
Steepest gradients search at (**a**) Murfreit and (**b**) Piz Boè sites. Resistivities are averaged over 10 m sections and plotted as a function of depth (blue line). The intersections with the vertical red lines are used to define the inflection points, i.e., the steepest gradient between the minimum and maximum resistivity values in the considered section. In Figure 7a, steepest gradients were not represented in the last 2 panels, since after 40 m the resistivity values are <<10^5^ Ohm m, and no ice presence is expected in the first 8 m of subsoil [50].

Note that ERT resistivity sections have been cut at a depth of 8 m—this, in order to be compared with the FDEM conductivity sections that cannot reach deeper information. The aim of the measurements was to verify the presence of the frozen soil and the ALT, defining also the boundary between unfrozen and frozen debris at the front of the rock glacier in the Murfreit site. In the Figure 5a and Figure 6a, resistivity values greater than 10^5^ Ωm were assumed as the presence of frozen material and presumed ice bodies [50]. In the Murfreit resistivity section (Figure 5a), it is clear that the highest values of resistivity are focused in the first 40 m of the survey line and the high resistivity layer tends to deepen toward the end of the line. The relative lower resistivity values in the ending part of the section in fact do not suggest the presence of frozen layers. To define the boundaries of the frozen/unfrozen zones, the steepest gradients method can be applied [77]. Figure 7a shows the resistivities (blue line), averaged along 10 m sections, for the Murfreit ERT inversion result and the second derivatives point between the lowest and greatest values (red lines). This way the boundaries between frozen and non-frozen debris can be suggested. The same procedure was adopted also for the Piz Boè inverted data (Figure 7b).

**Figure 8:**
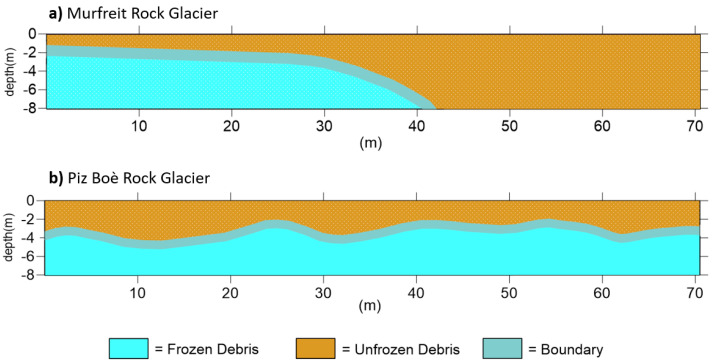
Ground interpretation in the investigation sites of (**a**) Murfreit rock glacier and (**b**) Piz Boè rock glacier, defined with the results of ERT and FDEM surveys. Boundary layer was suggested considering the uncertainties of the measurements.

This way, the ALT at the Murfreit site is estimated to range from 1.5 to 3 m in the first part of the section, while no frozen layer can be detected over 40 m distance [42]. Likewise, we can consider the resistivity section of the Piz Boè rock glacier. The presence of the frozen layer, along the survey line, can be estimated at a depth that varies between 2 m and 4 m. It is clear, as expected, that ERT investigations give us very informative results for the characterization of these rock glaciers. However, it is important to note that even the inversions of FDEM data allow us to obtain instructive conductivity sections (Figure 5b and Figure 6b). In fact, the FDEM-inverted profiles show the electrical-property distribution very similar to the corresponding ERT sections. In the conductivity section of the Murfreit rock glacier, Figure 5b, it is evident that lower conductivity values (<1 mS/m) are present only in the first 40–45 m of the investigation line, in agreement with the ground structure defined with the corresponding ERT survey. Even in the conductivity section of the Piz Boè site, Figure 6b, we can clearly see that lower conductivity values are at depths ranging between 3 m and 4 m, in agreement with the ALT estimated from the corresponding resistivity section. Consequently, with the presented results we can preliminarily assert that the two techniques provide very similar subsoil imaging. Figure 8 shows the final interpretation of the subsoil at Murfreit (a) and Piz Boè (b) sites, defined with the obtained results of ERT and FDEM investigations.

## 5. Discussion

The geophysical surveys conducted in the two sites of the Sella Group mountains provide useful information for the rock glaciers monitoring. The ALT and frozen/unfrozen boundaries can be in fact suggested both from ERT and FDEM results. Along the survey line of the Murfreit rock glacier, the ALT is around 2 m in the first part, quickly deepening to the fronts. After 40 m in fact, as suggested from FDEM and ERT data, no electrical values attributable to frozen soil are visible. This suggests that the front part of the Murfreit rock glacier is thawing with potential effect on dangerous debris releases, as observed by Krainer [42] and Mussner [78]. On the contrary, the measurement of the Piz Boè site suggests a constant ALT around 3–4 m, lying on a massive ice layer characterized by high-resistivity values. The Piz Boè results are in agreement with ERT surveys collected in 2011 [52] close to our investigated line, suggesting a steady condition for this rock glacier without the expected loss of ice due to temperature increase. The steady condition may be linked to the peculiar nature of this debris-covered glacier and to the morphology of eastern mountain face in comparison to the Murfreit one.

Comparing the ERT and FDEM sections, it is clear that conductivity values found with the FDEM inversion process are not directly comparable with the ERT resistivities in terms of absolute values. We tried also to calibrate the FDEM data with the ERT measurements; the distribution patterns do not change but the conductivities range, resulting from the inversion, go beyond the instrument resolution. This is due to the intrinsic instrumental resolution limit of the FDEM equipment in this very resistive environment. In fact, in the rock glaciers, the values of resistivity commonly span between 10^3^ and 10^6^ Ωm, so the response is close to the resolution limit of the FDEM instruments (for the CMD-Explorer ~0.1 mS/m). Furthermore, if compared to direct currents resistivity techniques, electromagnetic methods are more complicated, and the data are more sensitive to distortions. The propagation of the electromagnetic field in fact depends on more physical properties of the ground, i.e., magnetic, conductive, and dielectric properties. So, FDEM measurements have the great advantage of being quick and contactless but they are prone to more uncertainties, considering also that the transport of the device on debris blocks is not trivial. The operator in fact cannot guarantee the constant keeping of probe orientation and height from the ground. Scattered values, local anomalies or negative values of conductivity may be recorded due to a number of issues: electronic instrumental drift, heterogeneous height and orientation of the device, air temperature variations during the acquisition, variations of ground surface cover, lateral variations in grain size, near-surface variations in permafrost temperature or ice content, voids under rocks, presence of magnetic and/or metallic materials [71,75,79]. Consequently, in the rock glacier environment, the direct comparison of ERT and FDEM data should not be considered in terms of absolute values but rather in terms of electrical patterns [50,80,81], since the resistivity values obtained with these two different techniques may differ substantially.

It is the authors’ opinion that the FDEM technique provides very useful information for the mountain permafrost monitoring but cannot substitute borehole logging or other geophysical methods, such as RST or ERT. Thus, FDEM should be rather considered as an instructive method to extend, in a quick and convenient way, the information derived from the more accurate ERT technique. In fact, from a logistical point of view, it is practically impossible to cover, in a short time, a very large area of the rock glacier with ERT surveys. On the contrary, this is not true for the FDEM method. Hence, if a common investigation line has been acquired with these two techniques, this can serve as calibration transect to extend the information to the surrounding area with FDEM surveys. This way, the realization of 2D/3D mapping of the frozen/unfrozen boundary is possible. Nevertheless, it must be underlined that the challenging access to rock glacier environments can make dataset acquisition difficult, even with contactless methods. In our case study, for example, due to logistic timing problems with the helicopter’s transport, a 3D mapping of the areas was not possible and becomes a future research plan.

## 6. Conclusions

The Murfreit and Piz Boè rock glaciers were efficiently characterized, adopting both ERT and FDEM geophysical prospecting. The results obtained in our study cases confirm the well-known ability of the ERT technique to study the rock glaciers, but also the potentiality of the FDEM method.

FDEM provides a means to quickly acquire measurements that able to define the near-surface structure of these periglacial forms. The FDEM equipment is relatively light (CMD Explorer probe weighs 8 kg), and the measurements are collected simply by walking and carrying the device over the interested area, without the need for galvanic contact. This overcomes the high mountain environments’ access limits, where the heavy equipment of other geophysical surveys, such as ERT and RST, are more difficult to execute. Nevertheless, it must be noted that a rigorous acquisition protocol for FDEM measurements must be applied to avoid potential instrumental drift and, therefore, scattered values, local anomalies, or negative values of conductivity without physical meaning. From our field experience, this can be achieved by shortening the survey time to restrict air temperature changes, avoiding hottest daytime, and keeping the probe at constant height and parallel to the ground. Deep snow areas should be avoided, as wetter zones and metallic or magnetic materials, since they influence the measurements. Moreover, FDEM inversions do not provide as detailed and accurate results as ERT processing. For these reasons, the FDEM technique cannot be used individually but should be combined with other methods, such as borehole temperature data or ERT measurements, to efficiently extend the characterization in larger areas and create 3D subsoil models. The improvement of the acquisition skills and data processing suggests that FDEM can be used as a preliminary survey to quickly map wider areas of a rock glacier, defining the most interesting parts of where to perform more resolutive and deeper ERT investigations.

As for the FDEM measurements, the ERT method needs a rigorous approach to ensure the required resolution, penetration depth and the expected signal strength [50]. Reciprocal ERT acquisitions are strongly suggested since this allows one to evaluate the quality of the collected dataset and correctly define the expected data error of the inversion process, to minimize possible inversion artifacts. The major problem of the ERT technique in rock glacier environments remains the high contact resistances between the electrodes and rocky ground surface. In our experience, adding salt-water (300 g of NaCl per liter of H_2_O) between the electrodes and debris boulders is suggested to reduce this problem and avoid noisier datasets (about 0.3 l for each electrode of the array). To further improve the signal-to-noise ratio and obtain higher quality data, it is advisable to use as many stacks as possible, in our experience at least five. In the case of the noisier dataset (e.g., <50% quadrupoles saved after checking the reciprocals using the acceptable error threshold of 20%), it is preferable to carry out the inversion process by calculating a detailed error model, in order to use the entire dataset during the inversion process but assigning less weight to the quadrupoles with higher standard deviation. Finally, an efficient way to objectively define the sharp boundary between frozen and non-frozen debris, is to apply the steepest gradient method to the results of the ERT inversion.

Our future challenges will be the joint inversion of ERT and FDEM data, possibly by integrating RST measurements [82,83]. In such a way the inversion process can be constrained by more physical properties, increasing the confidence of our subsoil models [61].

## Figures and Tables

**Table 1:** ERT survey acquisition parameters.

**Instrument**	MAE Digital Georesistivimeter
**Power Supply**	60 A–12 V External battery
**Configuration**	Dipole–Dipole skip 0
**Current Injection Time**	250 ms
**Stack Max**	6
**V Min**	0.001 V
**V Max**	800 V
**Electrodes Number**	48
**Spacing**	1.5 m
**Array Length**	70.5 m

**Table 2:** Technical specifications of the multi-coil CMD Explorer FDEM (gf-instruments.cz).

Instrument Probe	Coil Spacing	Frequency	Nominal Exploration Depth (*HCP*–*VCP*)
1	1.48 m	10 kHz	2.2 m–1.1 m
2	2.82 m	10 kHz	4.2 m–2.1 m
3	4.49 m	10 kHz	6.7 m–3.3 m

## Data Availability

The data supporting the conclusions of this article will be made available by the authors on demand.
